# Season-long infection of diverse hosts by the entomopathogenic fungus *Batkoa major*

**DOI:** 10.1371/journal.pone.0261912

**Published:** 2022-05-05

**Authors:** Andrii P. Gryganskyi, Jacob Golan, Ann E. Hajek

**Affiliations:** 1 UES, Inc., Dayton, Ohio, United States of America; 2 Departments of Botany and Bacteriology, University of Wisconsin, Madison, Wisconsin, United States of America; 3 Department of Entomology, Cornell University, Ithaca, New York, United States of America; USDA Agricultural Research Service, UNITED STATES

## Abstract

Populations of the entomopathogenic fungus *Batkoa major* were analyzed using sequences of four genomic regions and evaluated in relation to their genetic diversity, insect hosts and collection site. This entomophthoralean pathogen killed numerous insect species from 23 families and five orders in two remote locations during 2019. The host list of this biotrophic pathogen contains flies, true bugs, butterflies and moths, beetles, and barkflies. Among the infected bugs (Order Hemiptera), the spotted lanternfly (*Lycorma delicatula*) is a new invasive planthopper pest of various woody plants that was introduced to the USA from Eastern Asia. A high degree of clonality occurred in the studied populations and high gene flow was revealed using four molecular loci for the analysis of population structure. We did not detect any segregation in the population regarding host affiliation (by family or order), or collection site. This is the first description of population structure of a biotrophic fungus-generalist in the entomopathogenic Order Entomophthorales. This analysis aimed to better understand the potential populations of entomopathogen-generalists infecting emerging invasive hosts in new ecosystems.

## Introduction

Species in the Entomophthorales are predominantly arthropod pathogens, serving important ecological roles ranging from modifying host behavior to regulating population dynamics [1–4]. However, host range among entomophthoralean species is poorly understood, complicated by limited information on species identities of both fungal pathogens and arthropod hosts. Moreover, advances in sequencing technologies have revealed the presence of several species complexes, resulting in what was once considered a species with multiple hosts in fact being several cryptic species with distinctive host specificities (e.g., the *Entomophaga aulicae* species complex [5], and the *Entomophthora muscae* species complex [6]). Additional complications arise when entomophthoralean-arthropod species combinations tested in the lab demonstrate pathogenicity, although field studies often reveal a narrower host range (i.e., ecological host range) than the lab host range (physiological host range [7]). Therefore, to understand the dynamics of diseases caused by entomophthoralean fungi in arthropod populations, it is critically important to identify the spectrum of potential arthropod hosts.

*Batkoa* is an excellent example of an entomophthoralean genus whose recent phylogenetic revision [8, 9] allows for careful comparisons with arthropod hosts. The genus was first described in 1989 [10] and now includes ten species [11]. Although at one point divided across six genera, recent phylogenetic studies provide parallel evidence that *Batkoa* is a single and distinct genus [9, 12]. Across all *Batkoa* species, the host range of *B*. *major* is among the best documented, with several reported host associations; in a worldwide compendium of entomophthoralean species, Bałazy [2] lists *B*. *major* as occurring in North and South America, as well as in Europe and Asia, and that it was “infecting several insect species of different orders,” including a ptilodactylid (Coleoptera), tipulids (Diptera), aphids (Hemiptera), and an ichneumonid (Hymenoptera). In 2018, *B*. *major* was also found alongside *Beauveria bassiana* (another entomopathogenic fungal species), co-infecting populations of the invasive spotted lanternfly (*Lycorma delicatula*, Fulgoridae, Hemiptera) [13]. This invasive fulgorid planthopper is only distantly related to native insects in the area of the co-epizootic (i.e., there are no native species in the same family, the Fulgoridae, in this area). At the time of the 2018 epizootics, *B*. *major* had only been cited from North America one time since its description in 1888 [8].

This study began with the goal of identifying the native reservoir hosts for *B*. *major*, a poorly known pathogen causing epizootics in outbreak populations of a new invasive insect. Based on trends in host range in the Entomophthorales, it was assumed that there would be few native host species and that these would predominantly belong to the order Hemiptera. We present results of a survey of naturally occurring infections in northeastern US forests that was conducted to identify hosts of *B*. *major*. We hypothesized that *B*. *major* in sampled locations is genetically diverse, but that it is not a species complex and that its populations are mostly clonal. Subsequent analyses investigated the genetic diversity and population structure of *B*. *major* to evaluate the potential for host specific clones and gene flow among collection sites.

## Materials and methods

### Sample collection and fungal isolation

Native insect populations were sampled in a mixed hardwood forest near Ithaca, New York. On nine days between 19 June and 14 September 2019, cadavers of insects killed by entomophthoralean fungi were collected in Danby State Forest, Tompkins County, New York. Collections were made along the Abbott Loop hiking trail between 42°18’56.3"N, 76°29’42.2"W and 42°17’44.9"N, 76°29’10.8"W. Native insects killed by entomophthoralean fungi were also collected along the borders of the Angora Fruit Farm (40°21’30.6”N, 75°53’00.4”W), Berks County Parks and Recreation, Pennsylvania on 19 September 2019, near *Ailanthus altissima* (tree of heaven; the preferred host tree for *L*. *delicatula*) and in the adjacent hardwoods. At both sites, all sides of leaves, twigs, and branches from the ground to 2.5 m were carefully surveyed for dead insects. Arthropod cadavers were placed in 29 ml clear plastic cups containing 5 ml of 1.5% water agar and were transported to the laboratory at 4°C. Collected insects were from low density populations of native species. Collection trips were made within 24–48 hours after rainfall and collections took place over a period of two to three hours per site. The two sample sites are approximately 220 km from each other.

In the laboratory cadavers that were sporulating or were ready to sporulate were moved to high humidity enclosures at room temperature. Each cadaver was separately covered with the base of a 60 mm petri dish containing malt extract agar (MEA; 30 g malt extract, 20 g agar, 1 L distilled water) to allow “ascending” conidia to be collected on the MEA [14]. After approximately 6 hours, petri dishes with conidia were removed. Cadavers that had not yet sporulated were left under high humidity at 20°C overnight and were irregularly checked for sporulation for a total of 48 h. After conidia had been collected, the body of each insect was stored at -20°C and subsequently examined to morphologically identify arthropod host species.

All MEA plates with conidia were maintained at 20°C. Once conidia had begun to germinate, thin sections of MEA containing hyphae were excised and placed in 35 mm petri dishes containing 1.5 ml 95% Grace’s insect medium (Lonza, Walkersville, MD) and 5% fetal bovine serum (Life Technologies, Grand Island, NY). Once hyphal growth was evident, hyphae were transferred to egg yolk Sabouraud maltose agar (EYSMA [14]) in 100 mm petri dishes. When cultures were mature, they were frozen in 10% glycerol in 2 ml cryotubes at -80°C, using a CoolCell Freezing System (Corning, NY) and deposited in the ARSEF culture collection (Table 1).

**Table 1:** Insect hosts collected in Pennsylvania and New York State, infected by *Batkoa major* in 2019–2020 (S1 File).

Collection		Host			ARSEF # (HLBio#)	GenBank accession #			
Site	Date	Order	Family or Suborder	Species		ITS1	ITS2	28S	*RPB2*
AFF	9/10 2018	Hemiptera	Fulgoridae	*Lycorma delicatula*	Bat13769	OL335101	OL335159	OL332699	OL624704
DSF	6/27/2019	Diptera	Rhagionidae	*	14421 (Bat42)	OL335043	OL335102	OL332659	OL624643
DSF	6/27/2019	Hemiptera	Cixiidae	*Cixius* sp.	14420 (Bat73)	OL335044	OL335103	OL332659	OL624644
DSF	6/27/2019	Coleoptera	Elateridae	*Athous brightwelli*	14426 (Bat83)	OL335045	OL335104	OL332659	OL624645
DSF	6/27/2019	Coleoptera	Cantharidae	*Rhagonycha fraxini*	(Bat86)	OL335046	OL335105	OL332637	OL624646
DSF	6/27/2019	Diptera	Dolichopodidae	*Gymnopterus* sp.	(Bat88)	OL335047	OL335106	OL332638	OL624647
DSF	6/27/2019	Coleoptera	Elateridae	*Athous brightwelli*	14444 (Bat90)	OL335048	OL335107	OL332639	OL624648
DSF	6/27/2019	Coleoptera	Tenebrionidae	*Isomira sericea*	(Bat91)	OL335049	**-**	OL332640	OL624649
DSF	7/10/2019	Diptera	Lauxaniidae	*Homoneura inserta*	(Bat120)	OL335050	OL335108	OL332641	OL624650
DSF	7/10/2019	Diptera	Sciaridae	*	(Bat121)	-	-	OL332642	-
DSF	7/10/2019	Diptera	Rhagionidae	*Symphoromyia* sp.	(Bat123)	OL335051	OL335109	OL332643	OL624651
DSF	7/10/2019	Diptera	Rhagionidae	*	(Bat124)	OL335052	OL335110	OL332644	OL624652
DSF	7/10/2019	Diptera	Rhagionidae	*	14427 (Bat132)	OL335053	OL335111	OL332645	OL624653
DSF	7/10/2019	Diptera	Rhagionidae	*	14430 (Bat156)	OL335054	OL335112	OL332646	OL624654
DSF	8/1/2019	Lepidoptera	Blastobasidae	*	14431 (Bat160)	OL335055	OL335113	OL332647	OL624655
DSF	8/1/2019	Lepidoptera	Tineidae	*Dryadaula* sp.	14448 (Bat163)	OL335056	**-**	OL332648	OL624656
DSF	8/1/2019	Lepidoptera	Erebidae	*Lophocampa caryae*	14435 (Bat164)	OL335057	OL335114	OL332649	OL624657
DSF	8/1/2019	Lepidoptera	Crambidae	*Eudonia* sp.	14437 (Bat165)	OL335058	OL335115	OL332650	OL624658
DSF	8/1/2019	Lepidoptera	Blastobasidae	*	14428 (Bat167)	OL335059	-	OL332651	OL624659
DSF	8/1/2019	Diptera	Rhagionidae	*	14432 (Bat173)	OL335060	-	OL332652	OL624660
DSF	8/1/2019	Diptera	Dolichopodidae	*Thrypticus* sp.	14436 (Bat174)	OL335061	-	OL332653	OL624661
DSF	8/1/2019	Diptera	Sciaridae	*	(179)	OL335062	OL335116	OL332654	OL624662
DSF	8/1/2019	Diptera	Sciaridae	*	14425 (Bat181)	OL335063	OL335117	OL332655	OL624663
DSF	8/1/2019	Diptera	Sciaridae	*	(Bat183)	OL335064	OL335118	OL332656	OL624664
DSF	8/1/2019	Diptera	Sciaridae	*	14433 (Bat184)	OL335065	OL335119	OL332657	OL624665
DSF	8/1/2019	Diptera	Sciaridae	*	14438 (Bat186)	OL335066	OL335120	OL332658	OL624666
DSF	8/1/2019	Diptera	Sciaridae	*	14449 (Bat189)	OL335067	OL335121	OL332659	-
DSF	8/7/2019	Coleoptera	Cantharidae	*Rhagonycha* sp.	14434 (Bat204)	OL335068	OL335122	OL332660	OL624667
DSF	8/7/2019	Diptera	Sciaridae	*	14439 (Bat205)	OL335069	OL335123	OL332661	OL624668
DSF	8/7/2019	Coleoptera	Cantharidae	*Rhagonycha* sp.	(Bat207)	OL335070	OL335124	OL332662	OL624669
DSF	8/7/2019	Hemiptera	Cixiidae	*Cixius* sp.	(Bat209)	OL335071	OL335125	OL332663	OL624670
DSF	8/7/2019	Lepidoptera	Blastobasidae	*	14424 (Bat210)	OL335072	-	OL332664	OL624671
DSF	8/7/2019	Coleoptera	Cantharidae	*Rhagonycha* sp.	(Bat217)	OL335073	OL335126	OL332665	OL624672
DSF	8/7/2019	Hemiptera	Derbidae	*Apache degeeri*	14457 (Bat220)	OL335074	OL335127	OL332666	OL624673
DSF	8/7/2019	Lepidoptera	Oecophoridae	*Fabiola edithella*	14440 (Bat221)	OL335075	OL335128	OL332667	OL624674
DSF	8/15/2019	Hemiptera	Cicadellidae	*	14441 (Bat222)	OL335076	OL335129	OL332668	OL624675
DSF	8/15/2019	Lepidoptera	Erebidae	*Lymantria dispar*	(Bat228)	OL335077	OL335130	OL332669	OL624676
DSF	8/15/2019	Hemiptera	Cicadellidae	*	14423 (Bat241)	OL335078	OL335131	OL332670	OL624677
DSF	8/15/2019	Hemiptera	Cixiidae	*Cixius* sp.	14422 (Bat242)	-	OL335132	OL332671	OL624678
DSF	8/15/2019	Diptera	Anthomyiidae	*	(Bat249)	OL335079	OL335133	OL332672	OL624679
DSF	9/4/2019	Diptera	Heleomyzidae	*Tephrochlamys rufiventris*	(Bat269)	OL335080	OL335134	OL332673	OL624680
DSF	9/4/2019	Psocoptera	Amphipsocidae	*Polypsocus corruptus*	(Bat270)	-	OL335135	OL332674	OL624681
DSF	9/4/2019	Diptera	Sciaridae	*	14450 (Bat271)	OL335081	OL335136	OL332675	OL624682
DSF	9/4/2019	Diptera	Psychodidae	*	14451 (Bat273)	OL335082	OL335137	OL332676	OL624683
DSF	9/4/2019	Diptera	Dolichopodidae	*Gymnopterus* sp.	(Bat275)	-	-	OL332677	-
DSF	9/4/2019	Hemiptera	Cixiidae	*Cixius* sp.	(Bat277)	-	-	-	OL624684
DSF	9/4/2019	Hemiptera	Achilidae	*	(Bat287)	OL335083	OL335138	OL332678	OL624685
DSF	9/4/2019	Hemiptera	Achilidae	*	(Bat288)	OL335084	OL335139	OL332679	OL624686
DSF	9/4/2019	Hemiptera	Achilidae	*	(Bat291)	OL335085	OL335140	OL332680	OL624687
DSF	9/14/2019	Diptera	Sciaridae	*	(Bat297)	OL335086	OL335141	OL332681	OL624688
DSF	9/14/2019	Hemiptera	Cixiidae	*Cixius* sp.	14458 (Bat300)	OL335087	OL335142	OL332682	OL624689
DSF	9/14/2019	Diptera	Sciaridae	*	(Bat301)	OL335088	OL335143	OL332683	OL624690
DSF	9/14/2019	Diptera	Sciaridae	*	14452 (Bat304)	OL335089	OL335144	OL332684	OL624691
DSF	9/14/2019	Diptera	Sciaridae	*	14453 (Bat309)	OL335090	OL335145	OL332685	OL624692
DSF	9/14/2019	Diptera	Sciaridae	*	14429 (Bat320)	OL335091	OL335146	OL332686	OL624693
DSF	9/14/2019	Diptera	Sciaridae	*	14454 (Bat321)	OL335092	OL335147	OL332687	OL624694
DSF	9/14/2019	Lepidoptera	Geometridae	*Lambdina fiscellaria*	(Bat322)	-	OL335148	OL332688	OL624695
DSF	9/14/2019	Diptera	Sciaridae	*	14455 (Bat323)	-	OL335149	OL332689	-
DSF	9/14/2019	Diptera	Sciaridae	*	(Bat324)	OL335093	OL335150	OL332690	OL624696
DSF	9/14/2019	Hemiptera	Derbidae	*Apache degeeri*	14459 (Bat326)	OL335094	OL335151	OL332691	OL624697
AFF	9/19/2019	Diptera	Sciaridae	*	(Bat327)	OL335095	OL335152	OL332692	OL624698
AFF	9/19/2019	Diptera	Lauxaniidae	*	(Bat332)	OL335096	OL335153	OL332693	-
AFF	9/19/2019	Diptera	Dolichopodidae	*Medetera* sp.	(Bat333)	-	OL335154	OL332694	OL624699
AFF	9/19/2019	Diptera	*	*	(Bat334)	OL335097	OL335155	OL332695	OL624700
AFF	9/19/2019	Diptera	Milichiidae	*Madiza glabra*	(Bat335)	OL335098	OL335156	OL332696	OL624701
AFF	9/19/2019	Psocoptera	Psocomorpha	*	(Bat340)	OL335099	OL335157	OL332697	OL624702
AFF	8/31/2020	Hemiptera	Fulgoridae	*Lycorma delicatula*	(Bat565)	OL335100	OL335158	OL332698	OL624703

AFF—Angora Fruit Farm, Berks County Parks and Recreation, Pennsylvania, DSF–Danby State Forest, Tompkins County, New York.–missing data,

*—not identified to the family/suborder, genus, or species level.

### DNA extraction and amplification

Fungal tissues from in vitro growth were transferred to lysis buffer and beaten with 0.5 g of 0.7 mm diameter zirconia beads at 4800 rpm for 1 min. DNA extraction and PCR were performed as described in Hajek et al. [15]. PCR was performed on 4 loci: 28S (large subunit of structural ribosomal RNA (rRNA) of eukaryotic cytoplasmic ribosomes), ITS1 and ITS2 (large subunit of structural ribosomal RNA (rRNA) of eukaryotic cytoplasmic ribosomes), and *RPB2*(large subunit of structural ribosomal RNA (rRNA) of eukaryotic cytoplasmic ribosomes). 28S amplification used forward primer LR0R [16] and reverse primer LR5 [17]. ITS1 amplification used forward primer ITS5 [18] and reverse primer 5.8S [17]. ITS2 amplification used forward primer ITS3 [18] and reverse primer ITS4sub: 5’-TGGAGCAAGTACAAACAACACT-3’. *RPB2* amplification used forward primer BatRPB2f: 5’- ACCCTCAGAAACCTCTCGTC-3’ and reverse primer BatRPB2r: 5’- CAAACCGAGCCAGCAATTTG-3’.

PCR conditions for 28S were initial denaturation for 5 min at 95°C followed by 6 cycles of denaturation for 1 min at 95°C, annealing at 58°C for 1 min that decreased by 1°C for each cycle, and extension for 1.5 minutes at 72°C. The 6 cycles were followed by 30 cycles of denaturation for 30 sec at 95°C, annealing at 52°C for 1 min, and extension for 1 min at 72°C. The final step was extension at 72°C for 10 sec. PCR conditions for ITSI and ITSII were an initial denaturation for 5 min at 94°C followed by 35 cycles of denaturation at 94°C for 45 sec, annealing at 55°C for 50 sec, and extension at 72°C for 1 min. The final step was extension at 72°C for 10 min. PCR conditions for *RPB2* were an initial denaturation at 95°C for 4 min followed by 34 cycles of denaturation at 95°C for 1 min, annealing at 50°C for 1 min, a ramp that increased the temperature at a rate of 0.3°C/sec for 1.23 min from 50 to 72°C, and extension for 1 min at 72°C. The final step was extension for 10 min at 72°C [19]. To check whether the PCR products were viable, products underwent agarose gel electrophoresis in 1x TAE buffer and were visualized with ACCURIS SmartDoc (Accuris, New Jersey, USA). Successful products were purified by combining 8μL of product with 2μL of a master mix (1.6μL molecular water, 0.2μL 10X PCR buffer, 0.1μL SAP enzyme, and 0.1μL EXO enzyme) at 37°C for 35 min followed by deactivating the enzymes at 90°C for 13 min. Purified PCR products were sequenced by Genewiz LLC (South Plainfield, New Jersey, USA). Sequences were edited, assembled, aligned, and searched using Geneious software v. 8.1.8 (Biomatters Ltd).

### Phylogenetic reconstruction and analysis of population structure

A single FASTA file was prepared from each of the four loci used to identify *B*. *major*: ITS1 (N = 39), ITS2 (N = 54), 28S (N = 66), and *RPB2* (N = 62). Each FASTA file was aligned using MAFFT version 7 (using default parameters with a scoring matrix of 1PAM/ *κ* = 2 for closely related sequences and was imported into R version 4.0.2 [20] for Maximum Likelihood inference and building a phylogenetic tree [21]. All analyses were conducted using the *adegenet* and *poppr* packages [22, 23]. Fungal samples in each FASTA file were further labelled according to the geographic location and arthropod host from which they were collected (accounting for host order, family, and species).

To infer the number of genetic clusters across our data set, and to evaluate the utility of arthropod host as a predictor of population structure in *B*. *major*, alignments of each locus were subjected to a Discriminant Analysis of Principle Components (DAPC) [24–26]. An additional DAPC was performed retaining only one sample per genotype per population using the clonecorrect command in *poppr* [23]. Population differentiation was further analyzed by calculating FST according to *B*. *major* host order and family using hierfstat [27].

## Results

In 2019, we collected a total of 213 insects that appeared to have been killed by entomophthoralean fungi. Most entomophthoralean species are difficult to isolate so collections resulted in a total of 67 samples of *B*. *major* (cultures plus fungus directly from cadavers) that could be used for molecular analysis.

### Morphological characterization

**Fig 1:**
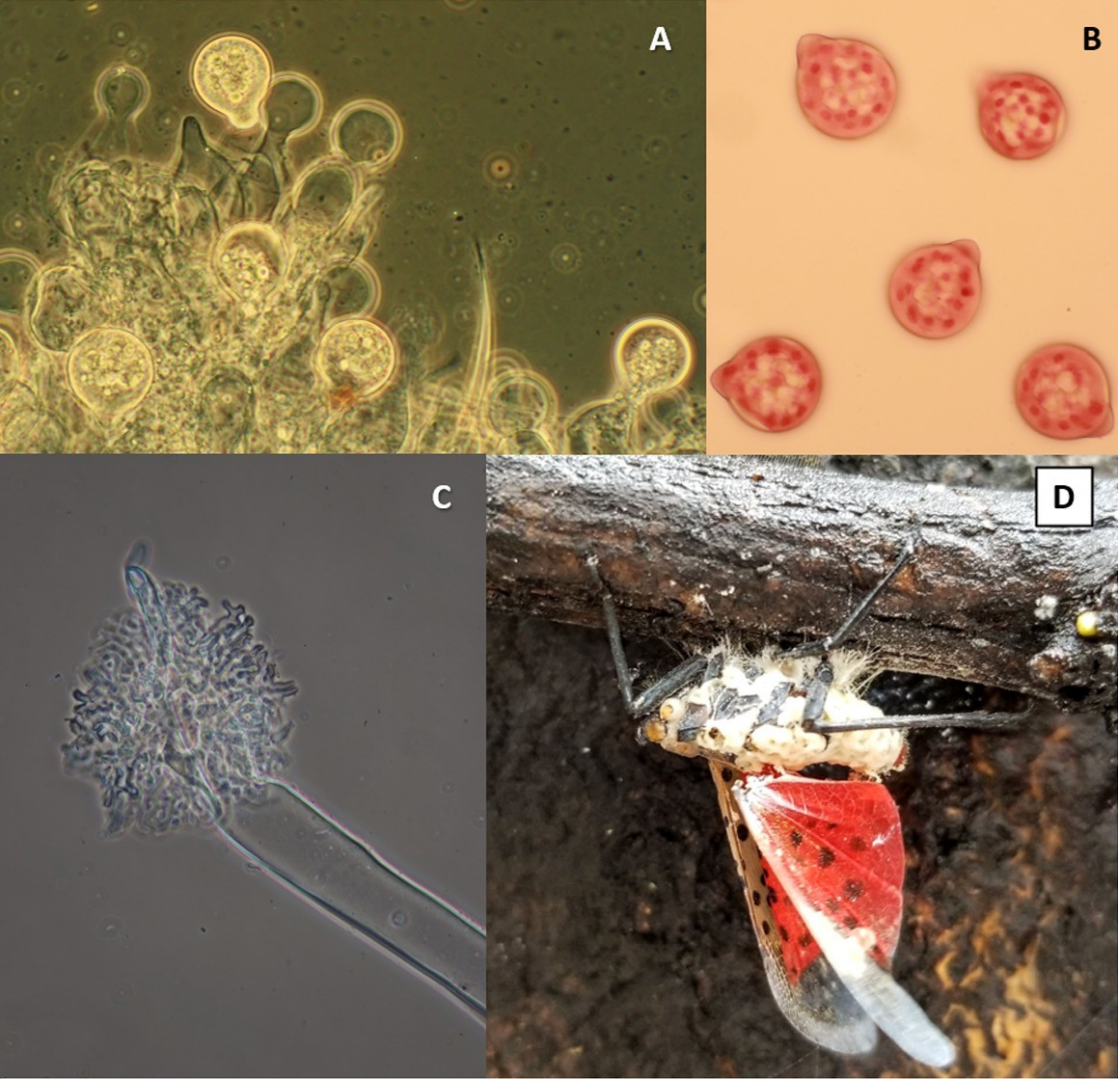
Micromorphology of *Batkoa major*. **A**. Conidiophores with conidia. Conidia average 41.5 μm wide x 49.3 μm long. **B**. Multinucleate conidia (nuclei stained with aceto-orcein). **C**. Distal end of rhizoid with holdfast. **D**. Cadaver of the spotted lanternfly attached to a twig by rhizoids (Photo by E.H. Clifton).

Our morphological observations of *B*. *major* from the cadavers of host insects do not differ from previously published records [2]. Diameters of conidia, size of conidial papillae, and the number of nuclei in conidia are typical for the species (Fig 1).

### Genetic polymorphism reveals the lack of host specificity

**Fig 2:**
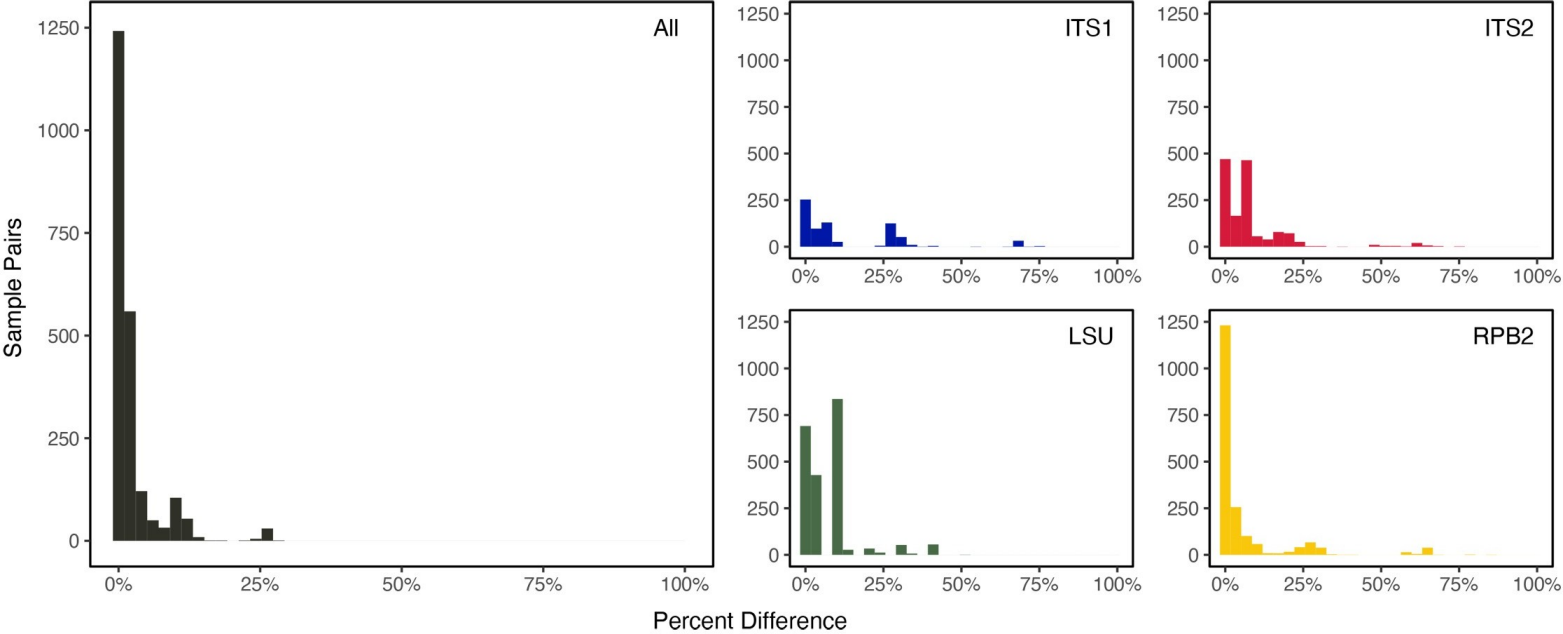
Percent genetic difference among pairwise comparisons of all specimens. Approximately half of all specimen pairs are identical for any given locus, as well as when all four loci are combined, suggesting that asexuality is an important aspect of the *B*. *major* life history. The least degree of polymorphism occurs in *RPB2*, whereas the most occurs in ITS1, in contrast to observations in other fungal species [28, 29].

Genotype comparisons and phylogenetic reconstructions suggests that these *B*. *major* populations consist of numerous genotypes. Alignments of four loci (28S, ITS1, ITS2 and *RPB2*) of *B*. *major* (S2 File) reveal a high degree of genetic polymorphism among individuals (Fig 2).

The combined alignments consisted of 3,201 positions (N = 67 specimens), with each locus presenting a varied degree of sites: 28S had a length of 1,067 bp with 20 polymorphic positions (N = 66), ITS1 had a length of 911 bp with 174 polymorphic positions (N = 39), ITS2 had a length of 639 bp with 58 polymorphic positions (N = 54), and *RPB2* had a length of 584 bp with 29 polymorphic positions (N = 62). Although we were unable to amplify and sequence the entire ITS region, and thus combine ITS1 and ITS2 sequences, we estimate the total length of the ITS region is greater than 1,600 nucleotides.

**Fig 3:**
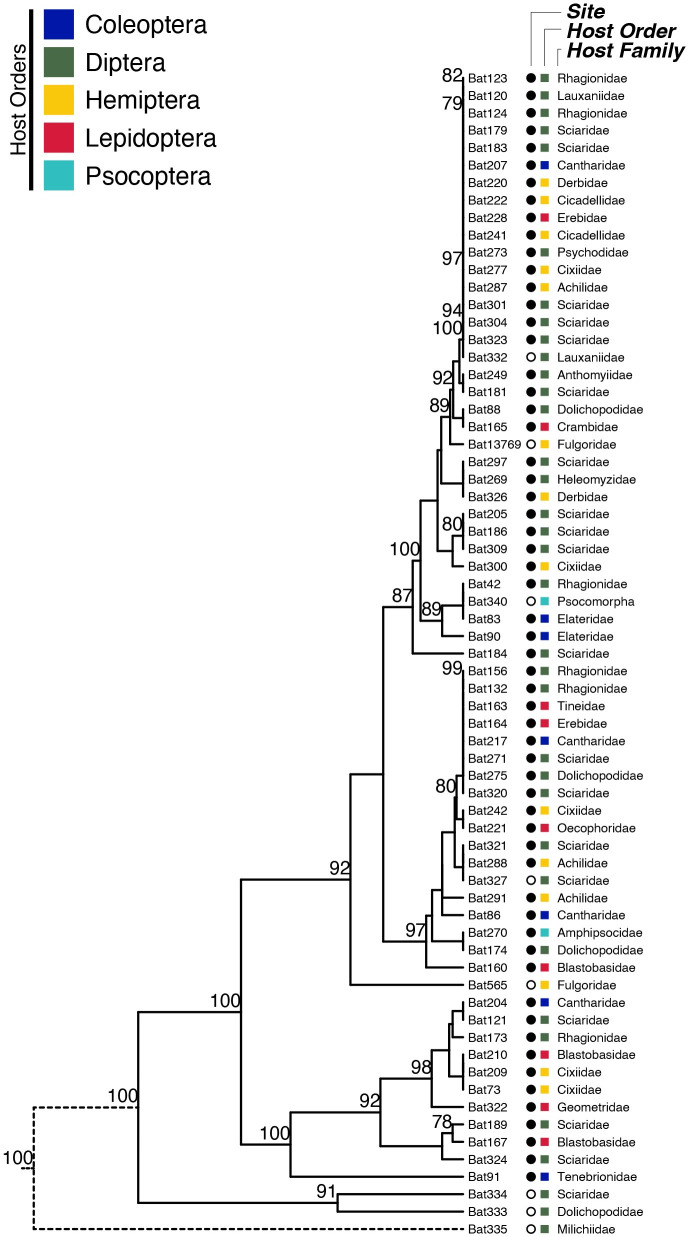
Dendrogram of all *B*. *major* isolates. Each specimen is labelled according to the order and family of the arthropod host from which it was sampled. Population origin is also specified, with an open circle for the Angora Fruit Farm population and a closed circle for the Danby State Forest population. Nodes whose bootstrap support was greater than 75 are labelled accordingly. A dotted black line was drawn to depict the outermost tree branch to indicate that it was shortened for aesthetic purposes.

Notably, genetic polymorphism does not correspond to arthropod host (Fig 3). For example, several clonal sample (i.e., those with branch lengths of zero in Fig 3) were collected from arthropod hosts distantly related to each other and belonging to different orders. Even when genetically dissimilar specimens are compared, several host orders or families are represented in a single clade.

Mapping hosts on the *B*. *major* phylogenetic tree demonstrated that hosts from the same family can be infected by pathogens that have different genotypes. There is no visible grouping of the hosts with particular clades of the pathogen, either on the single locus trees (S1 Fig) or on the combined four-locus tree (Fig 3). Major host orders and families are located on the *B*. *major* phylogenetic tree randomly. Slightly more than half of the infected insects belong to order Diptera (35 out of 67 samples). Among individual families, the most samples were from the Sciaridae (dark-winged fungus gnats; 19 out of 67 samples). Insect orders represented less frequently in the current screen are (in descending order): Hemiptera (N = 14 samples), Lepidoptera (N = 9), Coleoptera (N = 7), Psocoptera (N = 2).

**Fig 4:**
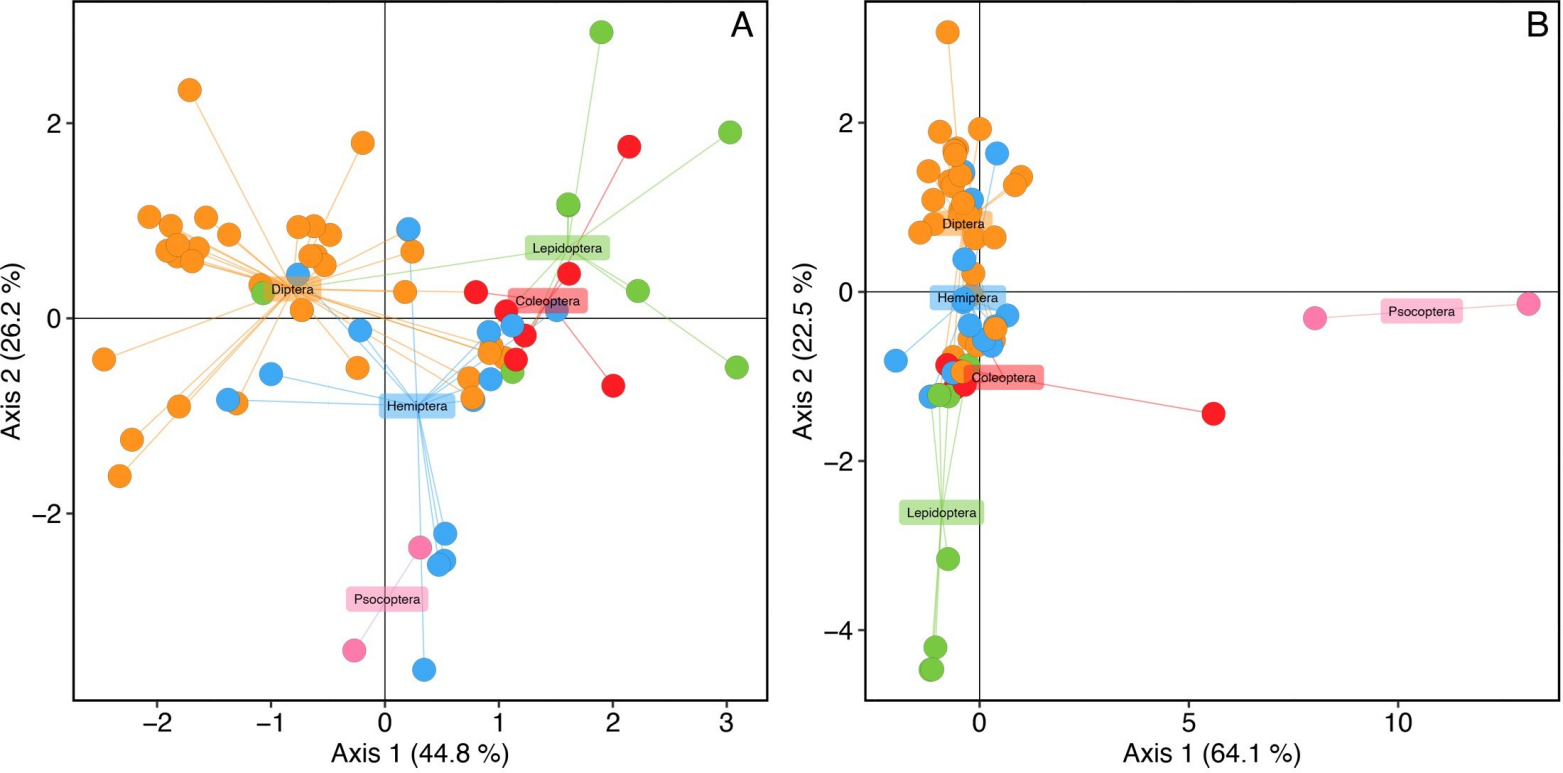
DAPC analysis for the combined ITS1, ITS2, LSU and *RPB2*. Data in full (**A**) and clone-corrected (**B**). Specimens are labeled according to host order. Axis 1 explained 44.8% (64.1% for clone-corrected data) and axis 2 explained 26.2% (22.5% for clone-corrected data) of the genetic variation among individuals.

Our DAPC analysis corroborates a lack of host specificity in *B*. *major*, illustrating a high degree of genotypic overlap when grouping samples by host order. The first (44.2% [64.1% clone corrected]) and second (26.2% [22.5% clone corrected]) principal components capture most of the genetic variation among samples and reveal no apparent clustering of individuals in correlation to their arthropod host (including when only a single specimen per genotype is retained in the analysis). An exception was the two samples from the Order Psocoptera that clustered together in the clone-corrected analysis (Fig 4).

### High gene flow within and between populations of B. major

Genotypes of *B*. *major* identified with either ITS1, ITS2, 28S or *RPB2* do not cluster according to collection site, in addition to not clustering by host order or family (Figs 3 and 4). Despite differences in sample size from our two collection sites, hosts were infected (often belonging to different arthropod orders and families) at each location by the same genotype, despite being separated by 220 km. Moreover, genetically distinct fungal specimens from the same population do not cluster phylogenetically, illustrating a broader pattern of high gene flow among genotypes of *B*. *major*.

**Fig 5:**
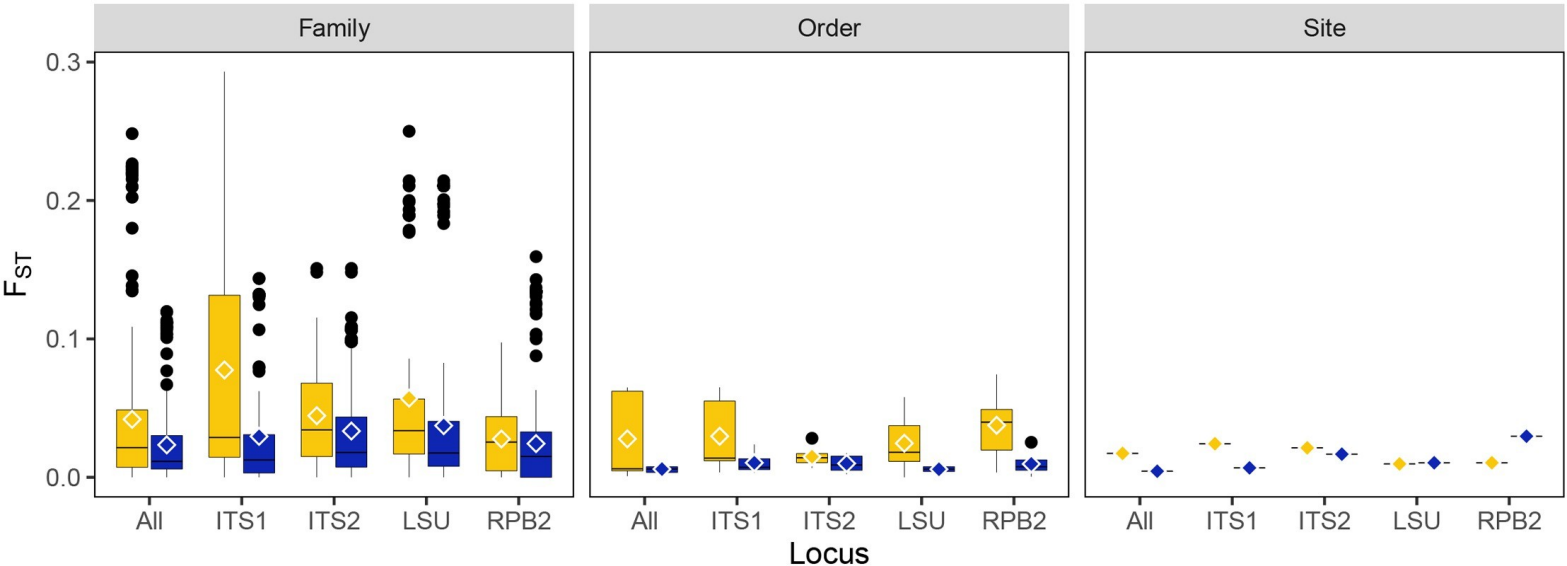
F_ST_ calculations for each locus and all four combined. F_ST_ calculated between host order, host family, and collection site (syn. population). Yellow–all data, blue–clone corrected data. Mean F_ST_ is illustrated by an open rhombus.

The lack of population structure by arthropod host is further supported by FST values calculated between host order and family, in addition to values calculated between the two collection sites (Fig 5). In all cases, median F_ST_ is 0.05–0.035 in all pairwise comparisons of host orders and families, with the maximum F_ST_ values approximately 0.20 when comparing host family by the 28S locus.

## Discussion

Entomophthoralean species have several different modes of host range diversity. *Batkoa major*, *B*. *apiculata*, *Zoophthora radicans*, and *Conidiobolus thromboides* have broader host ranges that include hosts in different insect orders. Then, there are some species of Entomophthorales with an intermediate type of host specificity, only infecting insects within the same insect order. For example, studies of physiological host range demonstrated that all three species in the *Entomophaga aulicae* species complex infect only species of Lepidoptera [30, 31]. Finally, there are highly specialized species, like *Strongwellsea magna* and *S*. *castrans*, that, even with extensive study, are known only from host species within individual families of Diptera [32]. The group of species with broad host ranges seems to be the smallest group within this fungal order [2]. Theory has suggested that more host specific parasites can lead to greater survival success [33].

Contrary to expectations, we found that the native fungal entomopathogen *B*. *major* has a diverse host range, including native insects in five insect orders. Why this fungal species has not been reported more previously is not known although one possibility is the difficulty of sampling insects in forested locations. Nonetheless, our findings agree with those for other fungal pathogens with broad host ranges, which are generally held to be more likely to form symbioses with novel hosts in invasive contexts [34, 35].

Invasive *L*. *delicatula* is thus a competent host for this native pathogen that is a generalist and which we found in abundance during a fall epizootic in *L*. *delicatula* [13] or all season long in different native host species. The well-known idiom ‘jack of all trades and master of none’ has been used to suggest that generalists would be less successful than specialists [33]. The alternate opinion that changes the idiom to ‘jack of all trades and master of all’ [36] is more consistent with results from the present study where *B*. *major* was found all season long infecting a diversity of hosts, although prevalence was not high in these lower density populations. Woolhouse et al. [37] suggest that conditions predisposing pathogens to generalism include high levels of genetic diversity as well as ample opportunities for cross-species transmission. While we found clonality for some groups of *B*. *major*, we found numerous clones including multiple samples from different hosts and gene flow occurred among some of them. We see some similarity in the population structure between *B*. *major* (our observations) and *E*. *muscae* [38]. In addition, our predominant collecting site was a native forest in New York during summer and the native insect fauna provided a diversity of hosts. Clones also existed within each of two clades of the entomophthoralean *E*. *muscae* infecting two species of flies [39].

Unusually rDNA sequence length significantly contributes to the polymorphism in our samples, possibly also due to higher substitution rates compared to other entomophthoralean fungi [9]. Curiously, the total length of the ITS region in *B*. *major* exceeds 1600 bp, which is quite an unusual feature compared to most fungal species. However, long ITS is also characteristic of other entomophthoralean species, e.g. for *E*. *muscae* [40] and *Zoophthora* species [41]. Also, genomes in some fungi contain multiple ITS copies [42]. Therefore, high population diversity in the *B*. *major* population recorded for the ITS1 and ITS2 regions might significantly reflect random ITS copies rather than real genetic diversity. The longer that the length of the ITS region is, the larger the number of mutations that might occur and therefore the larger number of different ITS copies that might be amplified and sequenced, which can be reflected as population diversity for that genomic region. In contrast, high numbers of identical copies suggest a high degree of clonality in the population, i.e., identical copies with different placement on the phylogenetic tree. It seems unlikely that we have randomly sampled the same ITS copy. This fact might be a good indication that the copies in *B*. *major* are homogenized by concerted evolution and sexual processes are occurring.

Interestingly, F_ST_ values indicate that not only is there high gene flow among populations, but also that there is high gene flow within populations vis-à-vis arthropod host. More specifically, our data not only indicate a lack of host specificity, but also that genotypes of *B*. *major* readily exchange genetic information (i.e., undergo sexual reproduction) with other genotypes infecting phylogenetically distant arthropod hosts.

Our finding of the same genotype of *B*. *major* at collection sites 220 km apart suggests that long-distance movement of *B*. *major* genotypes is occurring. Longer distance dispersal of *B*. *major* could be accomplished via dispersal of infected hosts. Insects can disperse longer distances and infected migratory aphids are known to provide dispersal of entomophthoralean fungi [43]. Most entomophthoralean fungi actively eject asexual spores which can result in airborne dispersal of these pathogens [1]. Such dispersal has been documented during epizootics for several species [44–46] and long distance dispersal has been modelled for *Entomophaga maimaiga* based on spread of this introduced pathogen [47].

Generalist pathogens are thought to potentially experience trade-offs in that they are not as well adapted to all the hosts that they infect [37]. While Bufford et al. [35] found that taxonomic similarity of co-evolved hosts with novel hosts was more important than contact opportunity, our study did not find any such patterns. In the present study, it could be possible that fitness could differ when *B*. *major* infects the invasive *L*. *delicatula* versus the diverse native hosts that were infected. Similar observations were made for the efficiency of *E*. *muscae* infecting even closely related muscoid species at the same location [48]. As opposed to the present study, the clones in *E*. *muscae* were associated with host species. For the fungal clavicipitacean genus *Metarhizium*, clones occurred within different species; however, because isolates came from soil samples, host relationships are not known [49].

Yet, even if individual fitness was decreased when *B*. *major* infected the novel invasive *L*. *delicatula*, being a generalist allowed *B*. *major* to take advantage of an outbreak population of an invasive host and we did not find native specialist pathogens responding to these outbreak invasive populations.

## Conclusion

The studied populations of *B*. *major* can infect various hosts in the same location. Analysis of molecular data supports the hypothesis of the clonal nature of the studied population. This can serve as a good example of a genetically diverse population of a pathogen-generalist with a certain amount of gene flow between its members. Use of a broad host range enabled *B*. *major* to switch to infection of the spotted lanternfly, a new invasive pest in the USA, which only appeared in Pennsylvania in 2014.

## Supporting information

S1 Fig Single locus trees. (PDF)

S1 File Underlying data. Molecular data on *Batkoa major*.
(XLSX)

S2 File Underlying data. *Batkoa major* alignment.
(TXT)
