# Supplementary material for: Season-long infection of diverse hosts by the entomopathogenic fungus Batkoa major
Source: PLoS One. 2022 May 5;17(5):e0261912. doi: 10.1371/journal.pone.0261912 (PMC9070890; doi:10.1371/journal.pone.0261912)
Supplement: S1 Fig — (PDF) [file pone.0261912.s001.pdf]

S1 Fig. Single locus trees

Absence of visible grouping of the hosts with particular clades of the pathogen on the single locus trees for ITS1 (**S1 A**), ITS2 (**S1 B**), 28S (**S1 C**), and *RPB2* trees (**S1 D**).

Fig. S1 A

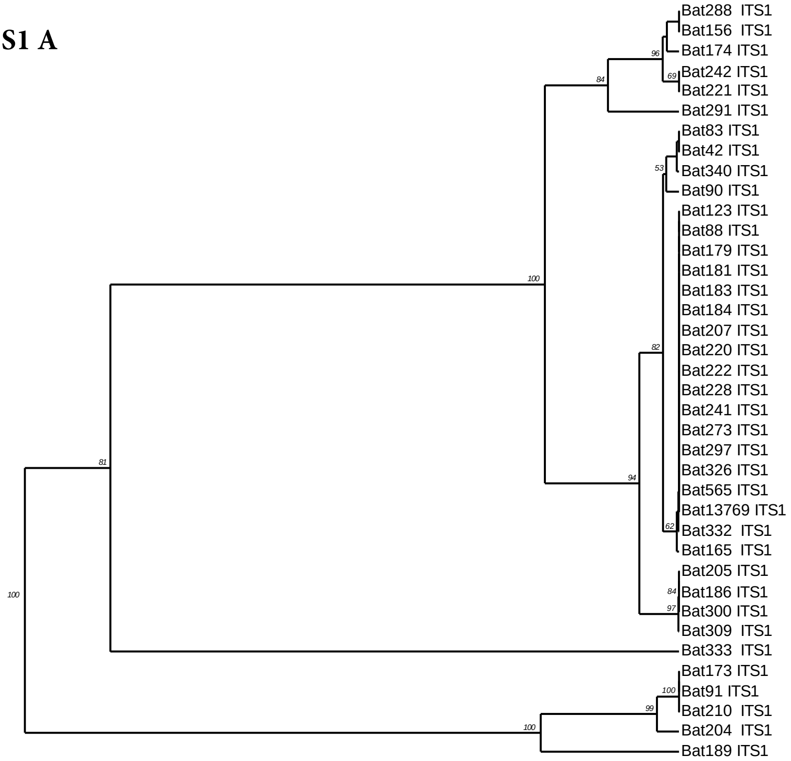

Fig. S1 B

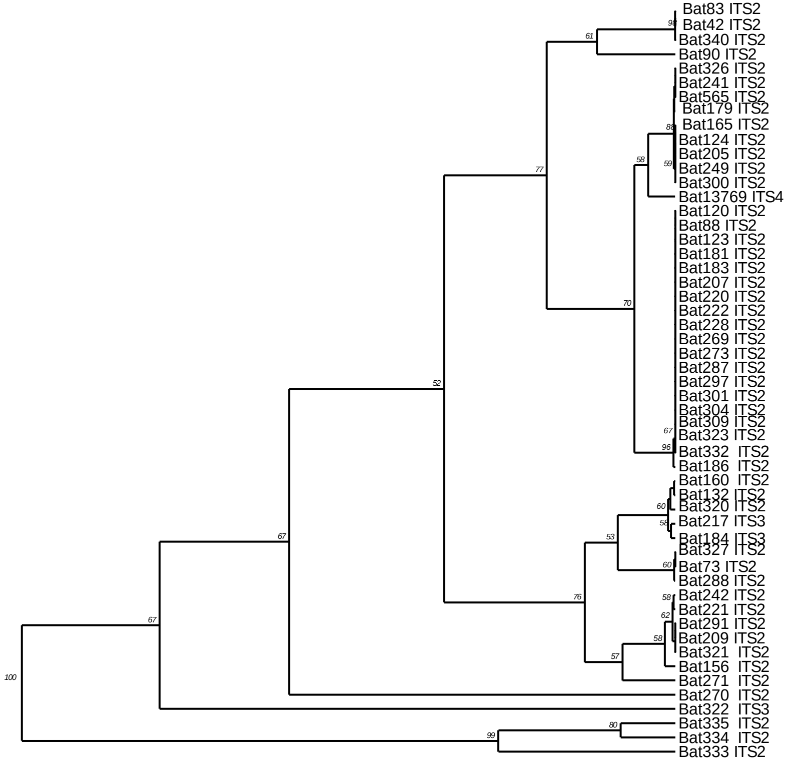
Bat83 ITS2
Bat42 ITS2
Bat340 ITS2
Bat90 ITS2
Bat326 ITS2
Bat241 ITS2
Bat565 ITS2
Bat179 ITS2
Bat165 ITS2
Bat124 ITS2
Bat205 ITS2
Bat249 ITS2
Bat300 ITS2
Bat13769 ITS4
Bat120 ITS2
Bat88 ITS2
Bat123 ITS2
Bat181 ITS2
Bat183 ITS2
Bat207 ITS2
Bat220 ITS2
Bat222 ITS2
Bat228 ITS2
Bat269 ITS2
Bat273 ITS2
Bat287 ITS2
Bat297 ITS2
Bat301 ITS2
Bat304 ITS2
Bat309 ITS2
Bat323 ITS2
Bat332 ITS2
Bat186 ITS2
Bat160 ITS2
Bat132 ITS2
Bat320 ITS2
Bat217 ITS3
Bat184 ITS3
Bat327 ITS2
Bat73 ITS2
Bat288 ITS2
Bat242 ITS2
Bat221 ITS2
Bat291 ITS2
Bat209 ITS2
Bat321 ITS2
Bat156 ITS2
Bat271 ITS2
Bat270 ITS2
Bat322 ITS3
Bat335 ITS2
Bat334 ITS2
Bat333 ITS2
61
77
58
59
70
67
96
52
60
58
53
60
76
58
62
58
57
67
67
100
80
99

Fig. S1 C

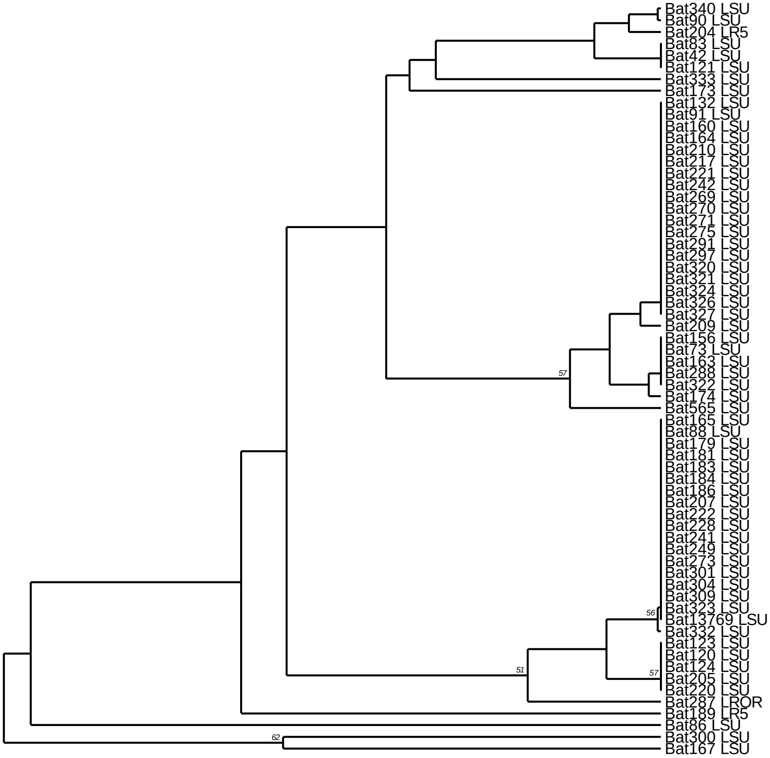

Bat340 LSU
Bat90 LSU
Bat204 LR5
Bat83 LSU
Bat42 LSU
Bat121 LSU
Bat333 LSU
Bat173 LSU
Bat132 LSU
Bat91 LSU
Bat160 LSU
Bat164 LSU
Bat210 LSU
Bat217 LSU
Bat221 LSU
Bat242 LSU
Bat269 LSU
Bat270 LSU
Bat271 LSU
Bat275 LSU
Bat291 LSU
Bat297 LSU
Bat320 LSU
Bat321 LSU
Bat324 LSU
Bat326 LSU
Bat327 LSU
Bat209 LSU
Bat156 LSU
Bat73 LSU
Bat163 LSU
Bat288 LSU
Bat322 LSU
Bat174 LSU
Bat565 LSU
Bat165 LSU
Bat88 LSU
Bat179 LSU
Bat181 LSU
Bat183 LSU
Bat184 LSU
Bat186 LSU
Bat207 LSU
Bat222 LSU
Bat228 LSU
Bat241 LSU
Bat249 LSU
Bat273 LSU
Bat301 LSU
Bat304 LSU
Bat309 LSU
Bat323 LSU
Bat13769 LSU
Bat332 LSU
Bat123 LSU
Bat120 LSU
Bat124 LSU
Bat205 LSU
Bat220 LSU
Bat287 LROR
Bat189 LR5
Bat86 LSU
Bat300 LSU
Bat167 LSU
57
56
51
57
62

Fig. S1 D

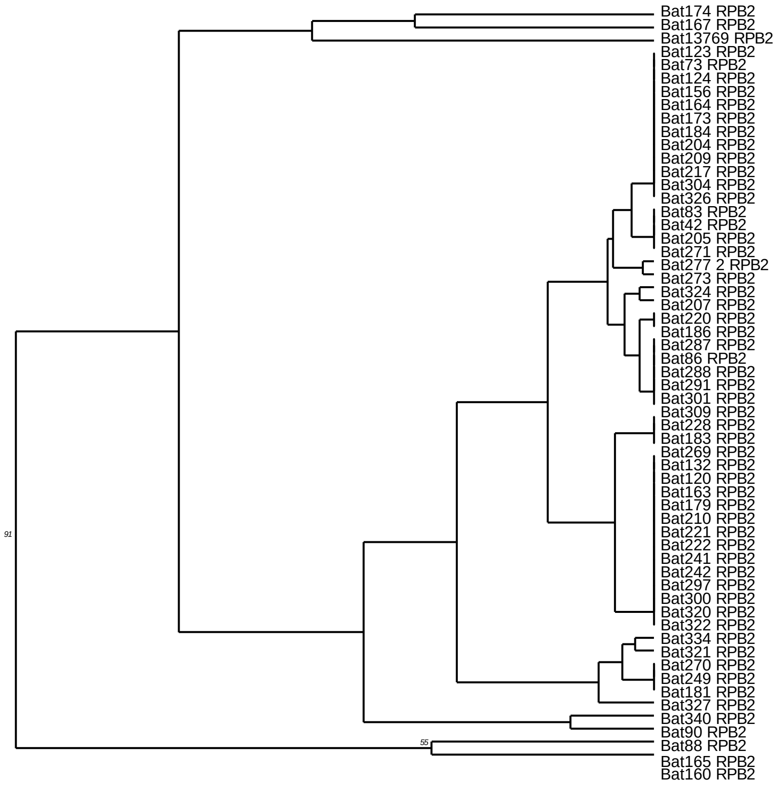

Bat174 RPB2
Bat167 RPB2
Bat13769 RPB2
Bat123 RPB2
Bat73 RPB2
Bat124 RPB2
Bat156 RPB2
Bat164 RPB2
Bat173 RPB2
Bat184 RPB2
Bat204 RPB2
Bat209 RPB2
Bat217 RPB2
Bat304 RPB2
Bat326 RPB2
Bat83 RPB2
Bat42 RPB2
Bat205 RPB2
Bat271 RPB2
Bat277 2 RPB2
Bat273 RPB2
Bat324 RPB2
Bat207 RPB2
Bat220 RPB2
Bat186 RPB2
Bat287 RPB2
Bat86 RPB2
Bat288 RPB2
Bat291 RPB2
Bat301 RPB2
Bat309 RPB2
Bat228 RPB2
Bat183 RPB2
Bat269 RPB2
Bat132 RPB2
Bat120 RPB2
Bat163 RPB2
Bat179 RPB2
Bat210 RPB2
Bat221 RPB2
Bat222 RPB2
Bat241 RPB2
Bat242 RPB2
Bat297 RPB2
Bat300 RPB2
Bat320 RPB2
Bat322 RPB2
Bat334 RPB2
Bat321 RPB2
Bat270 RPB2
Bat249 RPB2
Bat181 RPB2
Bat327 RPB2
Bat340 RPB2
Bat90 RPB2
Bat88 RPB2
Bat165 RPB2
Bat160 RPB2
91
55
